# Effect of Tillage System and Cover Crop on Maize Mycorrhization and Presence of *Magnaporthiopsis maydis*

**DOI:** 10.3390/biology9030046

**Published:** 2020-03-03

**Authors:** Mariana Patanita, Maria Doroteia Campos, Maria do Rosário Félix, Mário Carvalho, Isabel Brito

**Affiliations:** 1 MED—Mediterranean Institute for Agriculture, Environment and Development, Institute for Advanced Studies and Research, Universidade de Évora, Pólo da Mitra, Ap. 94, 7006-554 Évora, Portugal; mdcc@uevora.pt; 2 MED—Mediterranean Institute for Agriculture, Environment and Development & Departamento de Fitotecnia, Escola de Ciências e Tecnologia, Universidade de Évora, Pólo da Mitra, Ap. 94, 7006-554 Évora, Portugal; mrff@uevora.pt (M.d.R.F.); mjc@uevora.pt (M.C.); 3 MED—Mediterranean Institute for Agriculture, Environment and Development & Departamento de Biologia, Escola de Ciências e Tecnologia, Universidade de Évora, Pólo da Mitra, Ap. 94, 7006-554 Évora, Portugal; ibrito@uevora.pt

**Keywords:** arbuscular mycorrhizal fungi, late wilt, biotic stress, cover crop, extraradical mycelium, soil tillage

## Abstract

The sustainability of agriculture requires the adoption of agricultural soil conservation practices with positive impacts on soil quality, which can promote beneficial soil microbiota like arbuscular mycorrhizal fungi (AMF) and its diversity. This study aims to assess the influence of the presence of intact extraradical mycelium as a preferential source of inoculum of the native AMF in order to guarantee a better colonization as well as its possible bioprotective effect against *Magnaporthiopsis maydis*. In order to vary the available extraradical mycelium, two experiments, with and without cover crop, were carried out, in which two tillage systems and two maize varieties were studied. The capitalization of the benefits, in terms of grain production and *M. maydis* presence, associated to the cover crop were only achieved with minimum tillage. Therefore, both cultural practices are necessary to reduce the fungus presence, coupling the effect of mycorrhization together with other benefits associated with the cover crop. Although in the absence of a cover crop and using conventional tillage, yields and lower levels of *M. maydis* are possibly achieved, this system is more dependent on the variety used, does not benefit from the advantages associated with the cover crop, is more expensive, and environmentally unsustainable.

## 1. Introduction

Maize (*Zea mays* L.) is one of the most important cereal crops worldwide [1], with a global production amounting to more than 1100 Mt on about 194 million ha [2]. Currently, maize is predominantly produced in the United States of America, followed by China and Brazil. Maize exports are expected to grow by 19–157 Mt in 2027, the United States being the main exporter [3]. Maize plays a growing role in industry and energy resources. However, pests, diseases, and weeds have an impact on its yield and quality [4].

In recent years, late wilt disease caused by the soil-borne and seed-borne fungus *Magnaporthiopsis maydis* (Samra, Sabet, and Hing; Klaubauf, Lebrun and Crou [5], with the synonyms *Harpophora maydis*, *Acremonium maydis*, and *Cephalosporium maydis* (Samra, Sabet, and Hingorani)), morphologically and molecularly closely related to the Gaeumannomyces–Harpophora species complex [6,7], has emerged as an important disease of maize in the Iberian Peninsula [8], but has also been reported in India, Israel, and Hungary [9]. In infested fields, incidences up to 60% in susceptible varieties can cause 50% grain yield losses [9]. This disease is characterized by a rapid wilting of maize plants. Initial symptoms of maize wilt appear around the flowering stage, and, from tasseling to maturity, they steadily progress from the lower to upper leaves. As leaf wilting advances, yellowish or reddish-brown streaks appear on the basal internodes of the stalk, which dries up and shrinks [9]. Due to the delay in appearance of initial symptoms until about flowering, this disease has been designated as “late wilt” [10,11]. *M. maydis* is a soilborne vascular pathogen that penetrates the root tissues and colonizes the xylem [12]. Infection by *M. maydis* results in a reduced number of vascular bundles in the plants and in occlusion of xylem vessels by the growth of fungal mycelia [13]. The most effective way of controlling late wilt is the use of tolerant maize varieties [14,15]. Although the complete absence of symptoms in tolerant maize until the end of the crop season is not frequent, material displaying moderate to high tolerance should be included as an integrated strategy for the control of late wilt of maize [16]. Since the disease causes rapid and sudden wilting, an early diagnosis in plants is needed and may help to restrict disease spread. Due to the fact that infected seeds can carry the pathogen and spread the disease [17], molecular assays are important to recognize infected seeds and prevent spread to areas where the disease does not occur. In maize plants, the rot of the base of the affected stem and associated roots is partly due to secondary organisms (e.g., *Fusarium* spp.) from stalks primarily affected by late wilt [18].

Arbuscular mycorrhizal fungi (AMF) are an important component of the soil biota in most agroecosystems and colonize plant roots forming a mutualistic symbiosis. Arbuscular mycorrhiza (AM) likely made possible the conquest of land by the first bryophyte-like plants around 470 million years ago [19] and now colonize more than 80% of plants [20]. These obligate endotrophic symbionts are present across all soil types and biomes, in natural and anthropogenic ecosystems. According to current knowledge, these features are unique among other mutualistic symbioses and account for the pivotal importance of AM in earth’s ecosystem [21]. AMF comprise multinucleate and largely aseptate hyphae and are grouped in the phylum Glomeromycota [22]. 

Spores, fragments of AMF colonized roots and extraradical mycelium (ERM) are the possible inoculum sources, collectively termed propagules [20]. They are all able to start new mycorrhizal colonizations of plant roots, although the different propagule forms exhibit different colonization capabilities [23,24]. The ERM may be important for enhancing the roles of AM under field conditions considering root colonization from intact ERM starts earlier and develops faster than from other types of propagule [25,26], allowing the plant capitalization of benefits driven from mycorrhization earlier in the crop cycle [27,28]. When intact, ERM is such an effective propagule that even plant species that are usually poorly mycotrophic can be colonized when this propagule source is available [29]. Besides being an effective propagule source, when mycorrhization is installed, ERM plays several other important roles like the expansion of the soil volume used by the host plant, the enmeshing of soil particles, the connection of different plants (common mycorrhizal networks), and supports and interacts with soil biota. Additionally, ERM formed by indigenous AMF encompasses the functional diversity of the local fungal population and its associated microbes, which is expected to be greater than that of any introduced commercial inoculum. Under agricultural systems, ERM can develop on mycotrophic crops, cover crops [30], or natural vegetation that grows before seeding (weeds) [31] and can be kept intact if appropriate tillage techniques are used.

Many benefits can accrue to plants from their association with AMF, depending on the environmental conditions. In natural ecosystems, the most important role of AM may be in bioprotection rather than in the acquisition of nutrients [32]. The role of AMF in protecting their host against pathogens is well documented for several combinations of cultivated plants and fungal or nematode diseases [33]. Despite the complexity of all these interactions, it is recognized that a well-established AM is crucial for an adequate degree of protection [32]. The AMF must colonize the host plant and mycorrhization be well established before contact with the stressor, to achieve a high level of protection [34]. The large-scale inoculation with AMF is not a rational option in open field agriculture due to the high price of commercial inoculum, its lack of biodiversity, and weak persistence and efficacy. The development of management practices that maximize the benefits of the naturally occurring AMF inoculum, which is more biodiverse and adapted, seems to be a much better option to promote crop mycorrhization.

Tillage and crop rotation or the use of cover crops are key agronomic practices that need to be considered in developing sustainable production systems. The cultivation of crops that are natural hosts can increase the population of AMF [30] and thus help to maintain or increase mycorrhizal inoculum present in soil [35]. Tillage systems influence the physical, chemical, and biological environment of the soil, but their consequences for crop performance depend also on multiple interactions involving the soil, the climate, and the crop itself. Therefore, different tillage systems will have different effects on the crop performance. For AMF, the direct effects of the conventional tillage systems are related to physical disruption of the hyphal network and to the mixing of surface residues within the soil profile, also increasing the risk of soil erosion [36]. These can negatively impact the effectiveness of AMF, particularly the timing of colonization [27], because the ERM is broken and consequently the colonization is essentially initiated by sources of slow-growing inoculum (spores and colonized root fragments). When the ERM network integrity is affected there is a less efficient crop protection, due to the slower colonization of the plant by AMF [37]. Preserving the hyphal network created by the previous crop, through soil conservation techniques, will increase the ability of AMF to start the infection of the host plant [35], because the ERM remains intact. When host plants are present and the soil is not disturbed, hyphae from colonized roots and the soil mycelium network are the main source of mycorrhizal inoculum [24].

Since ERM colonizes earlier and develops faster than other sources of propagule, we hypothesized that AM formation starting from a well-established ERM from AMF and its associated microbial population, would provide a more efficacious protection against *M. maydis* in maize plants. The use of a cover crop to develop an ERM network, which can be maintained intact with a minimum tillage system, could be the way for the early AMF colonization of maize and, consequently, increase the potential bioprotective effect against *M. maydis*, which starts its colonization by slower growth inoculum forms. The goal of this study is to understand the potential associated to AMF through appropriate agricultural practices (cover crop and minimum tillage) in order to maximize the benefits provided by mycorrhization in the control of *M. maydis* in maize.

## 2. Materials and Methods

### 2.1. Study Sites and Experimental Design

**Figure 1:**
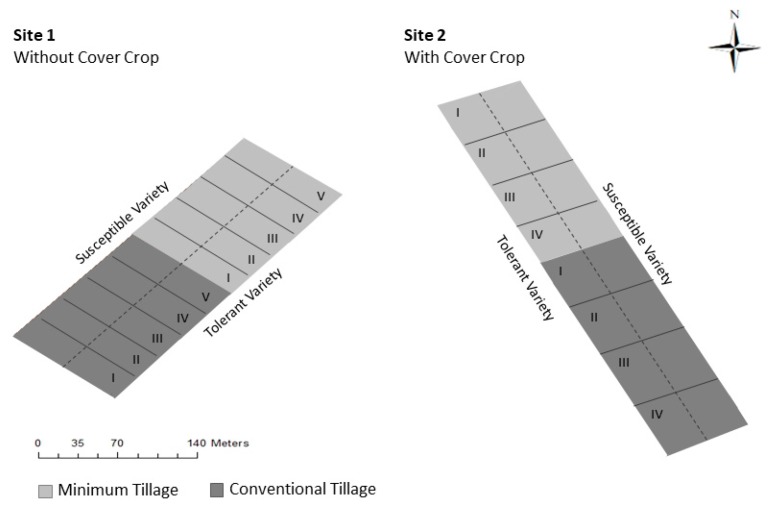
Experimental design of field experiments at site 1 and site 2. Two soil tillage systems (conventional tillage and minimum tillage) and two maize varieties (tolerant and susceptible to the fungus *Magnaporthiopsis maydis*) were considered, the roman numbers (I to V) represent the different replicates of each experiment.

Maize was used as the host plant and two field experiments were performed. The experiments were carried out with the collaboration of growers in two private farms of Ribatejo region (central Portugal), which were previously known to be infected with *M. maydis* for many years [38]. In one property (site 1) (39°20′11.738″ N, 8°32′59.978″ W), there was no cover crop, whereas in the other (site 2) (39°23′7.759″ N, 8°28′12.191″ W), a cover crop (*Lolium multiflorum*) was cultivated in the previous winter season. The experimental design in the field consists of four treatments with five and four replicates (site 1 and site 2, respectively), as shown in Figure 1, in which the soil tillage system (conventional tillage and minimum tillage), the maize variety (tolerant and susceptible to the fungus *M. maydis*), and the sampling date (two, four, and six weeks after emergence of the crop) were the study factors. The conventional agriculture system is based on inversion tillage, through the use of the moldboard plough and offset disks, and bailing of the cereal straw. On the other hand, minimum tillage is based on no soil inversion, using rigid or sprung tines that mobilize the soil only on the line. Harvest residues are left on the soil surface. The remaining technical itinerary was based on the usual procedures of each farm, and the crop was subjected to rain and irrigation, which was carried out through a conventional sprinkler irrigation system, according to the water needs in both experiments.

### 2.2. Sample Collection

Samples (biological replicates) consisted of maize roots and shoots. The samples of these two components were collected separately as bulked samples of six plants at three different times. Five or four replicates were considered in the sample collection (site 1 and site 2, respectively), two tillage systems (minimum and conventional tillage), two maize varieties (tolerant and susceptible to fungus *M. maydis*), and three sampling dates (two, four, and six weeks after emergence of the crop), which comprises a total of 60 samples collected in site 1 and 48 in site 2.

### 2.3. Parameters Evaluated

#### 2.3.1. Shoot Dry Weight (SDW)

After harvest, shoots samples were placed in paper bags that were then placed in a drying oven at 60 °C for approximately 72 h. After that period, the material contained in each sachet was weighed and the results of dry matter production (DM) were collected.

#### 2.3.2. Mycorrhizal Colonization

For each sampling date and treatment, root samples were stained with Trypan Blue. The staining procedure consisted of the following steps: (a) place about 0.7 g of roots of each composite sample in a histology cassette; (b) dip all cassettes into 10% (w/v) potassium hydroxide (KOH); (c) autoclave for 12.5 min at 121 °C to degrade and eliminate cellular constituents; (d) wash thoroughly with tap water to remove excess KOH and drain; (e) stain in a solution containing 0.1% Trypan Blue in lactoglycerol in the proportion of (1: 1: 1) (glycerol, 80% lactic acid and water) for about 11 min at 70 °C in a water bath. In this step, the Trypan Blue will bind to the chitin from the cell wall of the fungus; (f) remove the cassettes containing the stained roots from the solution described above and store in a 50% (v/v) glycerol solution. Roots were observed under the microscope after 48 h or remained submerged in the 50% (v/v) glycerol solution until further analysis.

To determine the mycorrhizal colonization, the intersection method described by McGonigle et al. (1990) was used [39]. In this method, the stained roots were mounted on microscope slides and covered with 24 × 60 mm coverslips. Roots were aligned parallel to the long axis of the slides and observed under an optical microscope at magnification ×200. For each sample, two slides were made and observed, both of which were treated as a single unit. The quantification of the mycorrhizal colonization was made by complete passes across each slide perpendicular to its long axis. The number of intersections of roots with vertical crosshair was counted in the following categories: “negative” (no fungal material in root), “arbuscules”, “vesicles”, and “hyphae only”. The arbuscular colonization (AC) and vesicular colonization (VC) were calculated by dividing the count for the “arbuscules” and “vesicles” categories, respectively, by the total number of intersections examined. Hyphal colonization (HC) was calculated as the proportion of non-negative intersections. Of all the possible forms of mycorrhizal colonization, only the presence of arbuscules and hyphae were considered, since these are representative of the degree of colonization of the root of a plant. All of the data collected using the magnified intersections method was examined in a random order with the identity of the roots unknown to the observer.

#### 2.3.3. qPCR quantification of *M. maydis* gDNA

Maize roots and *M. maydis* mycelium [38] were ground in liquid nitrogen and stored at −80 °C until further analysis. CTAB (hexadecyltrimethylammonium bromide) method was used to extract gDNA [40,41], and its quantification and evaluation of purity were determined using a NanoDrop-2000C spectrophotometer (Thermo Scientific, Waltham, MA, USA). gDNA integrity was evaluated by gel electrophoresis. Samples were diluted to a concentration of 20 ngμL^−1^.

A qPCR TaqMan assay for *M. maydis* was carried out in a 7500 Real Time PCR System (Applied Biosystems, Foster City, CA, USA) using 100 ng of gDNA as template, following the procedure previously described [38]. The quantification cycle (Cq) values, that are the PCR cycle numbers at which the reaction curve intersects the threshold line, inverse to the amount of target nucleic acids and correlated to the number of target copies, were acquired for each sample. gDNA from *M. maydis* was included in the analysis as positive control.

#### 2.3.4. Grain Production

Grain production was obtained from five and four replicates, respectively, at sites 1 and 2 of an area of approximately 612 m^2^ per replicate.

### 2.4. Statistical Analysis

Data analysis was performed using the MSTAT-C program (version 1.42; Michigan State University). The treatments were in factorial combination and the experimental design was a complete randomized block with five or four replicates (site 1 and site 2, respectively). The ANOVA analysis was carried out following the three-factor design: soil tillage system: “conventional tillage and minimum tillage” (two levels); maize variety: “tolerant and susceptible” (two levels) and sampling date: “two, four and six weeks after emergence of the crop” (three levels). Fisher’s Least Significance Difference (LSD) test was used to compare the means.

The ANOVA model of grain production in each field experiment was a two-factor (soil tillage system and maize variety). In order to be able to perform the joint analysis of grain production from both sites, a three-factor ANOVA (site, soil tillage system, and maize variety) was performed, for which data were transformed as a percentage of the maximum value of each experiment.

## 3. Results

The effect of the factors under study (soil tillage system, maize variety, and sampling date) for each of the parameters analyzed, namely hyphal colonization (HC) and arbuscular colonization (AC), dry matter (DM), and quantification cycle (Cq) values, indicators of *M. maydis* gDNA amount in qPCR reaction, were evaluated in each experiment. For the grain production, the influence of tillage system and maize variety was evaluated.

### 3.1. Site 1

In site 1, without cover crop, HC and AC were significantly affected by the three factors under study. Colonization rates were significantly greater for the minimum tillage and for the susceptible variety. Regarding the sampling date, both colonization rates increased, but not significantly, from date 1 to date 2, in which the highest values were obtained, and decrease significantly from date 2 to date 3. The DM was only influenced by sampling date, naturally increasing throughout the plant cycle. Cq values were not influenced by tillage system or sampling date. On the other hand, the tolerant variety showed a significantly higher Cq value than the susceptible variety (Table 1).

**Figure 2:**
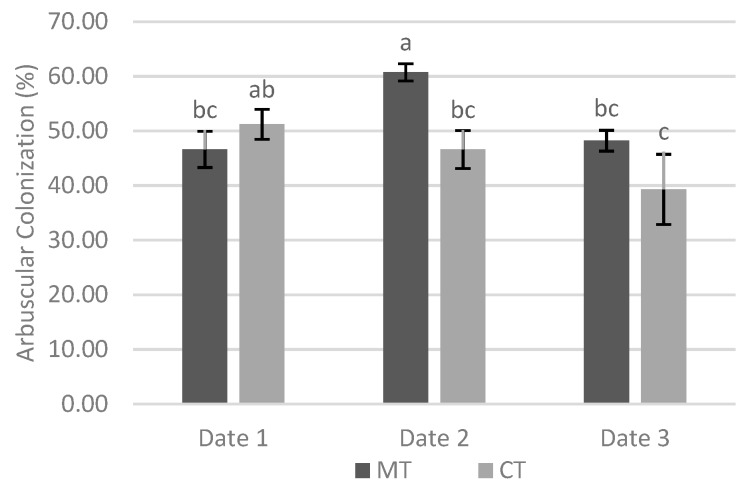
Effect of interaction between tillage system and sampling date in the arbuscular colonization on site 1. Different lowercase letters indicate significant differences for *p* < 0.05. Vertical upper bars represent the standard error of the mean. Caption: MT—minimum tillage; CT—conventional tillage.

The interaction between tillage system and sampling date was significant in relation to AC. At date 1 and date 3 there were no significant differences between two tillage systems, however at date 2 the AC was significantly greater under minimum tillage (Figure 2).

**Figure 3:**
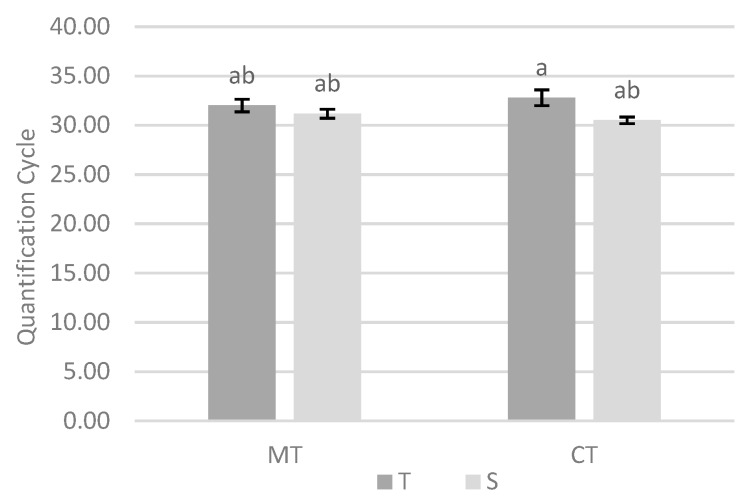
Effect of interaction between tillage system and variety in quantification cycle values on site 1. Different lowercase letters indicate significant differences for *p* < 0.10. Vertical upper bars represent the standard error of the mean. Caption: MT—minimum tillage; CT—conventional tillage; T—tolerant variety; S—susceptible variety.

The interaction between tillage system and variety was also reflected in significant differences in Cq values (Figure 3). Under minimum tillage there were no significant differences in the abundance of *M. maydis* between the two varieties. However, under conventional tillage, the tolerant variety differs significantly from the susceptible one, presenting a smaller amount of the phytopathogenic fungus.

Regarding grain production, it was influenced by tillage system, contrary to what happened in relation to the variety. Conventional tillage led to significantly greater grain production than minimum tillage (Table 1).

**Figure 4:**
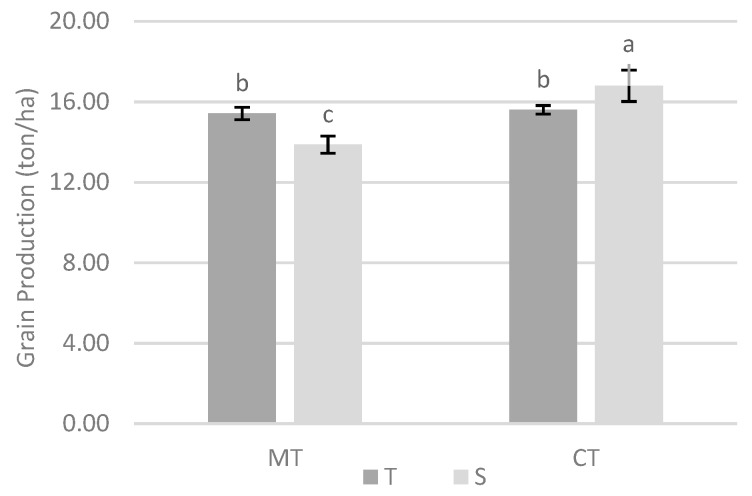
Effect of interaction between tillage system and variety in the grain production on site 1. Different lowercase letters indicate significant differences for *p* < 0.05. Vertical upper bars represent the standard error of the mean. Caption: MT—minimum tillage; CT—conventional tillage; T—tolerant variety; S—susceptible variety.

The interaction between tillage system and variety was significant, indicating that the variety production depended on the tillage system (Figure 4). Under minimum tillage, the tolerant variety produced more grain, whereas under conventional tillage, it was the susceptible variety that presented greater production. Although, overall, there were no differences in grain production between varieties, the analysis of the interaction tillage system × variety shows distinct behaviors.

### 3.2. Site 2

In site 2, with cover crop, HC and AC were significantly affected by variety and sampling date, showing higher values in the tolerant variety and at date 1. Regarding sampling date, it was verified that both colonization rates decrease significantly from date 1 to date 2 and increase, but not significantly, from date 2 to date 3. No effect of tillage system was observed in these parameters. The DM was influenced by tillage system and by sampling date, with greater values under minimum tillage and at date 3, and was not affected by variety. As expected, the DM increased significantly over three sampling dates. Cq values were only affected by tillage system, and with minimum tillage the Cq value was significantly greater than the observed with conventional tillage (Table 2), indicating a lower presence of *M. maydis*.

Although the difference is not significant, it should be noted that AC value on date 1 was greater under minimum tillage (data not shown).

**Figure 5:**
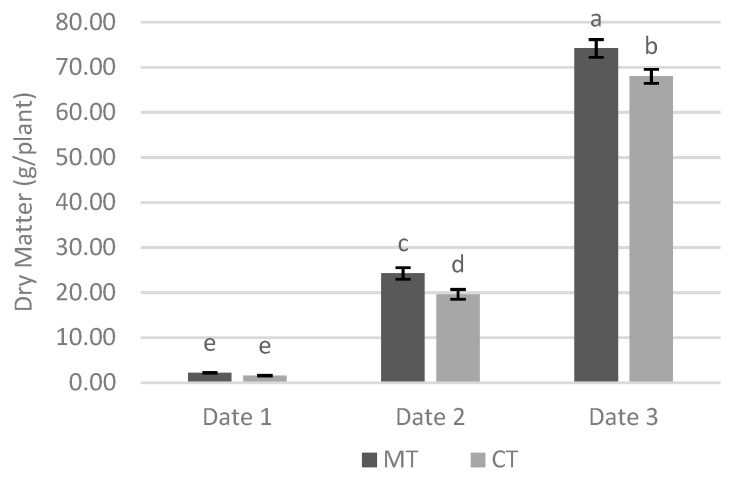
Effect of interaction between tillage system and sampling date in the dry matter production on site 2. Different lowercase letters indicate significant differences for *p* < 0.05. Vertical upper bars represent the standard error of the mean. Caption: MT—minimum tillage; CT—conventional tillage.

The DM, besides the effect of soil tillage and sampling date, was also significantly affected by the interaction between these two factors. Although on date 1, there were no significant differences between two tillage systems, on the two subsequent dates, minimum tillage presented DM values significantly greater than conventional tillage (Figure 5). It should be noted that despite no significant differences observed on date 1, the value of DM in minimum tillage was higher than in conventional tillage and this trend was intensified on dates 2 and 3, resulting in significant differences.

Regarding grain production, it was only influenced by tillage system (for *p* < 0.10). Minimum tillage led to significantly greater grain production than conventional tillage (Table 2).

### 3.3. Site 1 and Site 2

**Figure 6:**
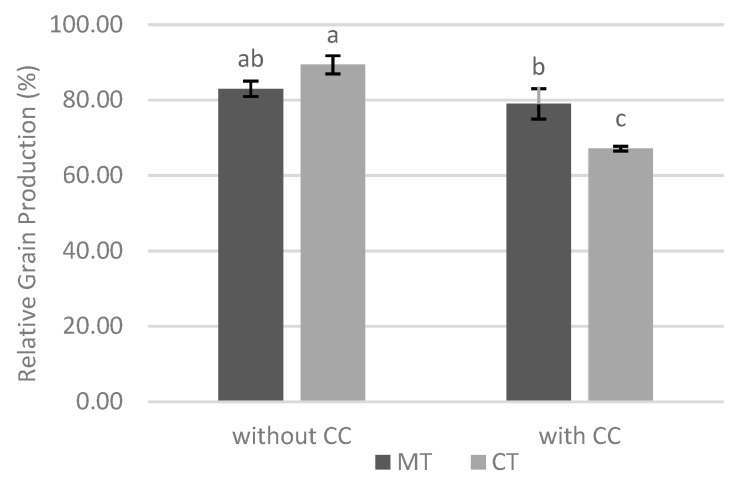
Effect of interaction between soil cover and tillage system in the relative grain production. Different lowercase letters indicate significant differences for *p* < 0.05. Vertical upper bars represent the standard error of the mean. Caption: MT—minimum tillage; CT—conventional tillage; CC—cover crop.

In order to compare the grain production of two sites, the production values were considered as a percentage of the maximum of each experiment, so that they were comparable. In the absence of cover crop, it was observed that there were no significant differences in relative grain production in both tillage systems. However, with cover crop, there was a significant advantage of minimum tillage (Figure 6).

**Figure 7:**
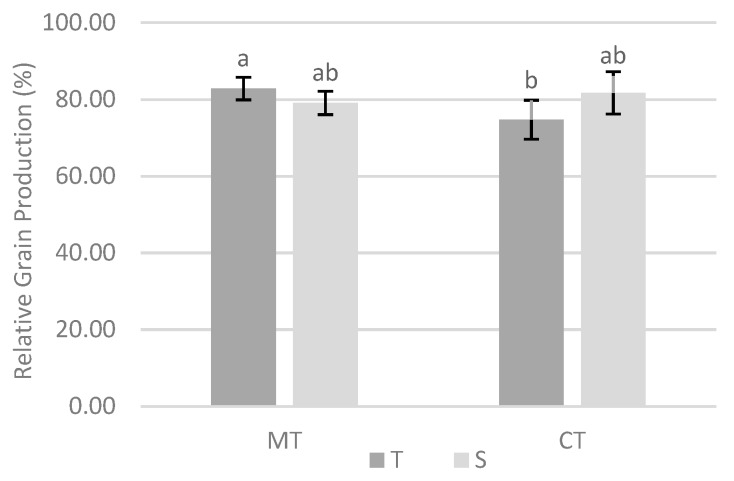
Effect of interaction between tillage system and variety in the relative grain production. Different lowercase letters indicate significant differences for *p* < 0.10. Vertical upper bars represent the standard error of the mean. Caption: MT—minimum tillage; CT—conventional tillage; T—tolerant variety; S—susceptible variety.

The relationship between tillage system and variety also influenced relative grain production (Figure 7). In minimum or conventional tillage, there were no differences in the relative grain production between tolerant and susceptible varieties. However, tolerant variety with minimum tillage leads to significantly higher relative yield than with conventional tillage.

**Figure 8:**
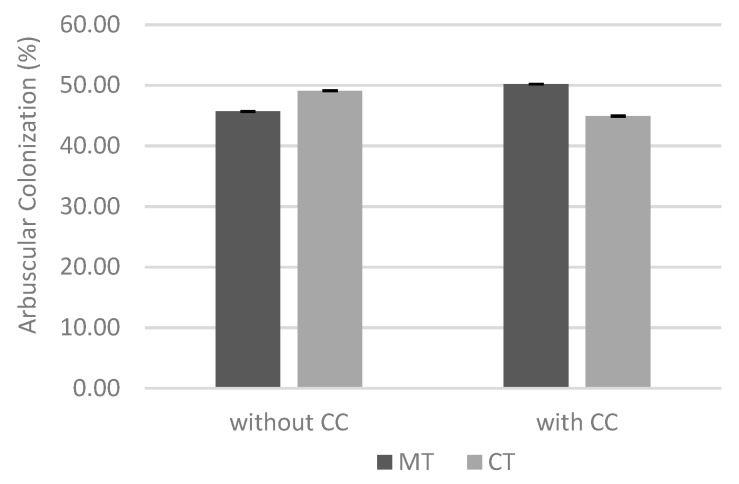
Effect of interaction between soil cover and tillage system in the arbuscular colonization at date 1. Vertical upper bars represent the standard error of the mean. Caption: MT—minimum tillage; CT—conventional tillage; CC—cover crop.

The results of AC were analyzed at both sites and for both tillage systems at date 1 (Figure 8). With cover crop and minimum tillage, the colonization is greater. Although there were no significant differences, the differences between two tillage systems were reduced in non-cover crop experiment. However, with cover crop, the differences between tillage systems are more pronounced, and minimum tillage leads to a higher colonization rate of maize.

**Figure 9:**
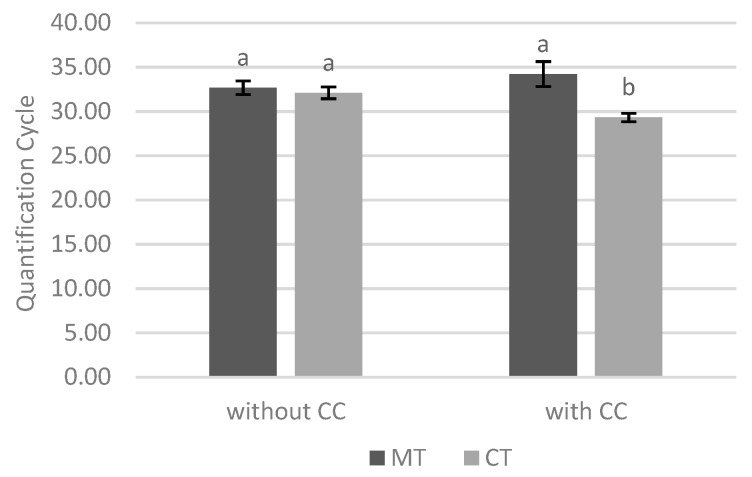
Effect of interaction between soil cover and tillage system in quantification cycle values at date 1. Different lowercase letters indicate significant differences for *p* < 0.05. Vertical upper bars represent the standard error of the mean. Caption: MT—minimum tillage; CT—conventional tillage; CC—cover crop.

The results obtained for Cq values at both sites and tillage systems at date 1 were also analyzed (Figure 9) and it was observed that, without cover crop, there were no differences in the amount of the fungus for both tillage systems. In contrast, with cover crop, minimum tillage led to a significantly lower amount of *M. maydis* in the plants when compared to conventional tillage.

## 4. Discussion

Considering the results obtained in field experiments, it was possible to verify different levels of *M. maydis* infection due to the production techniques used, as well as different levels of colonization by AMF and variations in dry matter and grain production. Even if no cover crop is used, the weeds that were in the field before sowing the maize can be themselves mycotrophic. Minimum tillage, because it did not destroy the ERM developed by this natural vegetation, allowed significantly higher rates of arbuscular and hyphae colonization of maize (Table 1). Although the mycelium developed by the weeds was eventually less abundant, it was found that when maintained intact it had a positive effect on maize colonization by AMF [42]. It was also verified that colonization rates were higher in the susceptible variety, just as Cq value was significantly lower for this variety (Table 1), that is, the abundance of phytopathogenic fungus in this variety was higher, as expected. Since the selection of varieties used was based only on phenotyping tests in relation to the visible symptoms of the disease, and the mechanisms of tolerance involved are unknown, it is not known to what extent they interfere with the process of mycorrhizal colonization. The fact that both colonization rates (by hyphae and arbuscules) are higher in the susceptible variety and the abundance of *M. maydis* in this variety is also higher, leads us to suppose that the entry mechanisms of AMF and phytopathogenic fungus may not be competitors. In this case, there is no effect of mycorrhization in the abundance of *M. maydis.* However, the lower amount of *M. maydis* in the tolerant variety had no effect on dry matter production (Table 1). It is important to note that the symptomatology of this disease is only noticeable at an advanced stage of the vegetative cycle [43] and since samplings were performed at two, four, and six weeks after emergence, the effect that the presence of the fungus could cause in the production of dry matter at this stage was not yet visible. It was also observed that the dry matter production increased over time (Table 1), as expected.

Mycorrhizal colonization rates, in general, decreased over time (Table 1). This fact happens since the colonization rates simultaneously integrate the effect on the growth of the two partners of the symbiosis: the plant and the AMF. Thus, a reduction in colonization rates may not mean unfavorable conditions to AMF growth, but only be the result of a further growth of the plant root system. One way to overcome this issue is to use colonized root density (CRD), which integrates root and AMF growth, allowing a three-dimensional evaluation of the progression of symbiosis and evolution of the total number of arbuscules per unit of colonized root length and per unit of volume of soil. This parameter may discriminate the effect of growth conditions on each of the symbionts [44]. However, CRD evaluation involves measuring the root system length of the host plant, which in a field experiment of this nature would be impractical. In the present study, it is probable that the presence of AMF has been diluted by a greater development of the root, since maize is a spring/summer irrigated crop that responds easily and quickly to the inputs that it is supplied and, therefore, show a great root growth.

In relation to arbuscular colonization, the interaction between tillage system and sampling date was significant (Figure 2), and on date 1 and date 3 there were no significant differences between the two tillage systems; however, at date 2 the arbuscular colonization was significantly higher with minimum tillage. The high colonization rates observed since the first sampling date reveal that this was an already late time point and compromised the perception of eventual differences in the early colonization of the culture, imposed by the factors under study. In fact, at the first sampling, the colonization rates observed were already relatively high, and the initial dynamics of colonization may not have been fully covered.

Without cover crop and conventional tillage, tolerant variety was significantly different from susceptible variety, presenting a smaller amount of *M. maydis* (Figure 3). In this case, it can be said that genetics is an effective tool in reducing the damage caused by late wilt in maize. However, with minimum tillage the differences between maize varieties in relation to the amount of *M. maydis* were no longer observed, which emphasizes the need to use different cultural practices, namely the use of a minimum tillage system, which allow a lower pathogenic fungus abundance, regardless of the variety used.

Regarding grain production, the values obtained in the experiment without cover crop were significantly greater under conventional tillage (Table 1). However, there was an interaction between tillage system and maize variety, which indicates that the behavior of variety depended on the type of soil tillage. It was verified that, with minimum tillage, the tolerant variety produced more grain, whereas, with conventional tillage, it was the susceptible variety that had the highest production (Figure 4). Although there were no differences in grain production at the variety level, the analysis of the interaction tillage system × variety shows distinct behaviors. In fact, while tolerant variety did not give rise to significantly different yields, regardless of tillage system, this was not the case with susceptible variety, which was significantly more productive with conventional tillage, although this was also the situation in which there was a higher amount of *M. maydis* (Figure 3). Apparently, in these circumstances, the higher amount of phytopathogenic fungus associated with the susceptible variety did not translate into a decrease in grain production.

The use of a mycotrophic cover crop allows for the development in the soil of an extensive network ERM which, if maintained intact by non-tillage or reduced soil tillage, can provide early colonization of the subsequent crop and thus better protect it against any biotic or abiotic stresses [21]. At site 2, with cover crop, there are no significant differences in mycorrhizal colonization rates (HC and AC) with minimum or conventional tillage (Table 2), as would be expected in the presence of a mycotrophic cover crop. This is likely related to the selection of the first sampling date, which could have been too late. This was the first work performed with a population of native AMF in maize in this region. Although there is already some knowledge in this scientific area for wheat [45], it is a winter crop with lower growth rates. Despite the anticipation of the first sampling date when compared to wheat experiments, it has proved to be inaccurate, therefore an even earlier sampling will be required in future work to detect the eventual very initial mycorrhizal colonization differences.

Contrary to the experiment of site 1 without cover crop, in site 2 mycorrhizal colonization rates were higher for the tolerant variety (Table 2). Knowing that AMF are not specific to certain plant hosts but have preferential associations [46] it might be possible that the AMF assemblage developed in association with the cover crop is also preferential for the tolerant maize variety. It may also be because at site 2 the planting occurred more than one month later than at site 1 and as such the temperature conditions at the initial stage of the culture were different, conditioning the development of the AMF and the crop. In addition, there are no differences between varieties regarding the presence of *M. maydis* (Table 2), which leads us to think that the advantages associated with the cover crop overlap the effect of the variety. In this case, and contrary to what happened in the previous experiment, the Cq value is significantly greater under minimum tillage (Table 2). Keeping in mind that conservation agriculture is based on no-till, maintenance of crop residues on the soil surface and crop rotation [47,48], these results highlight the need to increase the use of conservation agriculture practices to reduce the incidence of the disease. When a mycotrophic cover crop is used, beside many other benefits, it allows the development of the ERM, that if kept intact, will promptly colonize the following crop. By conserving this well-developed ERM using minimum tillage, maize roots come into contact with the inoculum source that will provide an early, faster, and more intense colonization [27,45,49], so that the plants will benefit both the absorption of nutrients and protection against biotic or abiotic stresses, since the early stages of the vegetative cycle. This colonization also offers the possibility of early activation of plant defense mechanisms, both locally and systemically [50] and eventually better face the *M. maydis* threat.

With cover crop, dry matter production was significantly greater under minimum tillage than under conventional tillage (Table 2), which can be due to the benefits associated with cover crop [35], which translated into advantages for maize at the level of its development, but probably also due to the significantly less amount of *M. maydis* present under these circumstances. This behavior of maize showed the benefit of the presence of an intact ERM previously developed by the cover crop.

With cover crop, it was also found that colonization rates generally declined over time, as previously reported, and that dry matter production increased over time (Table 2), as expected. Concerning dry matter production, the interaction between tillage system and sampling date did not show significant differences between two tillage systems on date 1. However, on the two subsequent dates differences were found, with minimum tillage having significantly higher values than the conventional tillage (Figure 5). The minimum tillage, because the experiment has a cover crop, presents an advantage to the conventional tillage. Since the ERM developed in the soil by the cover crop remains intact, this contributes to a better development of the crop [51].

The sustainability of agricultural activity is closely related to the way soils are managed [21,52]. Therefore, adopting conservation practices, such as the use of cover crops and minimum tillage systems, can positively affect crop productivity. The grain production values obtained in the experiment with cover crop are thus significantly higher for minimum tillage (Table 2). In this way, it was verified that, with cover crop and minimum tillage, maize presented a higher dry matter yield, a higher grain yield, and a lower amount of *M. maydis* in its roots, which clearly shows the benefits of using the combination of cover crop and minimum tillage.

Comparing the two experiments in terms of grain production, it was found that without cover crop, there were no significant differences in relative grain production between the two tillage systems. However, with cover crop, grain production value was closer to the maximum with minimum tillage (Figure 6). This system only presents an expressive translation in the increase of the local production when associated with a cover crop, which seems to be related to the effect of the cover crop and all the associated benefits, as well as the presence of an intact ERM of mycorrhizal fungi. The capitalization of the benefits derived from the cover crop, including the reduction of *M. maydis* in maize plants, was better achieved with minimum tillage, so it makes sense to use this soil tillage system when a cover crop has been previously installed. Comparing two sites under study (with and without cover crop), and although there were no significant differences for varieties in each tillage system (Figure 7), the tolerant variety yielded significant higher grain yield with minimum tillage. This result seems to be associated with the expected comparative advantage of the use of a tolerant variety and minimum tillage system that led to higher Cq values (lower amount of *M. maydis*), mainly with cover crop. Thus, in the environments in which the phytopathogenic fungus is present, the importance of conservation cultural practices (cover crop and minimum tillage) stands out, which may be associated with the use of a tolerant variety. However, under the specific conditions of the experiments, there were no significant differences in relative grain production between varieties, and therefore it appears that the use of the tolerant variety has no advantage.

Although there were no significant differences at date 1, it was found that with cover crop and minimum tillage, the arbuscular colonization was higher (Figure 8). Thus, in the presence of cover crop and when the ERM was maintained intact, acting as the preferred source of inoculum, the arbuscular colonization of maize roots at an early stage was larger and developed faster, which is in agreement with previous work [26,27,45,49]. ERM network integrity can be affected when there is soil disturbance, thus reducing plant colonization by the AMF and, consequently, providing less efficient crop protection [37]. With conventional tillage there is a reduction in the colonization by AMF once the ERM is broken, so colonization is essentially initiated by sources of slow-growing inoculum. Therefore, the best way to guarantee and achieve good initial colonization rates of the crop is to avoid the destruction of the ERM network of the native and naturally biodiverse inoculum, using soil conservation techniques [21].

It was also verified that, without cover crop, there were no differences in the presence of phytopathogenic fungus when comparing two tillage systems. On the other hand, with cover crop, the plants cultivated with minimum tillage were less infected with *M. maydis* than the ones cultivated with conventional tillage (Figure 9). Thus, in order to reduce the presence of *M. maydis* fungus in maize, two cultural practices, use of cover crop and minimum soil tillage system, should be associated, bringing together the beneficial effect of mycorrhization (more indirectly detected in this study) and other benefits associated with the cover crop. In these circumstances, the use of tolerant or susceptible varieties seems to be indifferent.

## 5. Conclusions

The presence of a well-developed intact ERM, using a cover crop and minimum tillage, is a strategy with benefits for the crop both in its growth and in the protection against biotic stresses, namely in protection against *M. maydis*. As noted, one of the limitations to the use of AMF in agricultural ecosystems is the idea that the time elapsed is high so that a sufficient level of colonization of the host plant is achieved to guarantee bioprotection of the crop. However, if symbiosis is well established from the beginning of the vegetative cycle, which is achieved by using the type of propagule that promotes an early and faster colonization, intact ERM, it is possible to overcome this challenge. It is crucial to have better knowledge of the AM symbiosis. Many farmers are unaware of AM or that their benefits are within reach and can be obtained by the adoption of simple crop management techniques, bypassing the need to use commercial inoculum. In conclusion, in environments where *M. maydis* is known at the outset, the choice of cultural conservation practices is particularly important for maize cultivation, namely the installation of a cover crop and minimum tillage system. Although in the absence of a cover crop and using conventional tillage it is possible to reach interesting yields, this system is more dependent on the maize variety used, does not benefit from the advantages associated with the cover crop, besides the negative impacts associated to conventional tillage like for example soil erosion, and decrease of soil organic carbon or energy demand (more powerful machinery).

## Figures and Tables

**Table 1:** Hyphal and arbuscular colonization (HC and AC), dry matter (DM), quantification cycle (Cq), and grain production values obtained for each factor under study on site 1. For each parameter, different lowercase letters indicate significant differences for *p* < 0.05. SE: Standard error of the mean.

Treatment	HC (%) (±SE)	AC (%) (±SE)	DM (g/plant) (±SE)	Cq Value (±SE)	Grain Production (ton/ha) (±SE)
***Soil Tillage***					
Minimum Tillage (MT)	54.40 (±1.91) a	51.80 (±1.77) a	14.64 (±3.42) a	31.58 (±0.48) a	14.64 (±0.36) b
Conventional Tillage (CT)	48.60 (±2.62) b	45.70 (±2.67) b	16.20 (±3.56) a	31.64 (±0.49) a	16.20 (±0.43) a
***Variety***					
Tolerant (T)	48.60 (±2.54) b	45.80 (±2.58) b	15.51 (±3.48) a	32.39 (±0.28) a	15.51 (±0.18) a
Susceptible (S)	54.30 (±2.02) a	51.70 (±1.91) a	15.33 (±3.50) a	30.83 (±0.29) b	15.33 (±0.64) a
***Sampling Date***					
Date 1	50.60 (±2.17) ab	48.90 (±2.15) ab	4.14 (±0.16) c	32.39 (±0.50) a	
Date 2	57.30 (±2.48) a	53.70 (±2.47) a	18.33 (±0.55) b	31.52 (±0.32) a	
Date 3	46.40 (±3.37) b	43.70 (±3.41) b	48.71 (±0.99) a	30.92 (±0.69) a	

**Table 2:** Hyphal and arbuscular colonization (HC and AC), dry matter (DM), quantification cycle (Cq), and grain production values obtained for each factor under study on site 2. For each parameter, different lowercase letters indicate significant differences for *p* < 0.05. ^1)^ Significant differences for *p* < 0.10. SE: Standard error of the mean.

Treatment	HC (%) (±SE)	AC (%) (±SE)	DM (g/plant) (±SE)	Cq Value (±SE)	Grain Production (ton/ha) (±SE)
***Soil Tillage***					
Minimum Tillage (MT)	39.90 (±2.60) a	38.00 (±2.54) a	33.55 (±6.32) a	33.36 (±0.87) a	11.03 (±0.65) a^1)^
Conventional Tillage (CT)	41.80 (±2.02) a	39.30 (±1.88) a	29.73 (±5.88) b	30.48 (±0.36) b	9.38 (±0.56) b^1)^
***Variety***					
Tolerant (T)	45.20 (±1.71) a	42.80 (±1.66) a	31.98 (±6.16) a	31.97 (±0.76) a	9.73 (±0.62) a
Susceptible (S)	36.50 (±2.53) b	34.60 (±2.41) b	31.29 (±6.08) a	31.87 (±0.70) a	10.69 (±0.69) a
***Sampling Date***					
Date 1	50.40 (±2.12) a	47.70 (±2.13) a	1.89 (±0.10) c	31.77 (±0.96) a	
Date 2	34.90 (±2.32) b	33.80 (±2.25) b	21.92 (±1.02) b	31.47 (±0.65) a	
Date 3	37.30 (±2.45) b	34.60 (±2.29) b	71.09 (±1.45) a	32.52 (±1.04) a	
